# Disease Control With FOLFIRI Plus Ziv-aflibercept (zFOLFIRI) Beyond FOLFIRI Plus Bevacizumab: Case Series in Metastatic Colorectal Cancer (mCRC)

**DOI:** 10.3389/fonc.2019.00142

**Published:** 2019-03-14

**Authors:** Wafik S. El-Deiry, Arthur Winer, Michael Slifker, Stanford Taylor, Blythe J. S. Adamson, Neal J. Meropol, Eric A. Ross

**Affiliations:** ^1^Department of Hematology, Oncology, Fox Chase Cancer Center, Philadelphia, PA, United States; ^2^Warren Alpert Medical School, Providence, RI, United States; ^3^Biostatistics and Bioinformatics Facility, Fox Chase Cancer Center, Philadelphia, PA, United States; ^4^Population Studies Facility, Fox Chase Cancer Center, Philadelphia, PA, United States; ^5^Flatiron Health, New York, NY, United States

**Keywords:** ziv-aflibercept, FOLFIRI, mCRC, colorectal cancer, metastasis, disease control, zFOLFIRI

## Abstract

**Background:** The prognosis of patients with metastatic colorectal cancer (mCRC) is poor, especially after failure of initial systemic therapy. The VELOUR study showed modestly prolonged overall survival (OS) with ziv-aflibercept plus 5-fluorouracil, leucovorin, and irinotecan (zFOLFIRI) vs. placebo+FOLFIRI after progression on 5-fluoruracil, leucovorin, and oxaliplatin (FOLFOX) ± bevacizumab. The utility of zFOLFIRI after bevacizumab+FOLFIRI is unknown and not recommended in NCCN guidelines. We explored whether zFOLFIRI may be active beyond progression on bevacizumab+FOLFIRI.

**Methods:** We undertook a retrospective analysis of patients treated in routine clinical practice. A chart review was conducted for a cohort (*N* = 19) of advanced cancer patients (18 mCRC) who received zFOLFIRI from 2014 to 2018 at Fox Chase Cancer Center (FCCC). Analysis included time on zFOLFIRI, PFS, OS, CEA trends and adverse events. A second mCRC cohort (*N* = 26) from the Flatiron Health EHR-derived database treated with zFOLFIRI after prior bevacizumab+FOLFOX and bevacizumab+FOLFIRI was analyzed for time-on-treatment and overall survival.

**Results:** Median age of mCRC cohort at zFOLFIRI treatment was 54 (FCCC; *N* = 18) and 62 (Flatiron Health-cohort; *N* = 26). Of 18 FCCC mCRC patients, 1 patient had prior bevacizumab+FOLFOX and ramucirumab+irinotecan prior to zFOLFIRI for 8.5 months. Of 17 FCCC mCRC patients with prior bevacizumab+FOLFIRI who received zFOLFIRI, 13 had mutant-KRAS, 3 WT-KRAS, and one BRAF-V600E. The patient with BRAF-V600E mutation achieved stable disease on zFOLFIRI after multiple BRAF-targeted therapies. One patient (WT-KRAS mCRC) remained on zFOLFIRI for 14 months. Of 14 patients with mutated-KRAS, 8 remained on zFOLFIRI for >5 months including 3 for >15 months. The rate-of-change in CEA measures on zFOLFIRI was significantly different (*p* = 0.004) between rapid progressors and those with PFS>4 months. For mCRC patients treated with zFOLFIRI in the 3rd line or greater (*N* = 18), median PFS was 7.1 months (214 days) and median OS was 13.8 months (416 days). Median time-on-treatment with zFOLFIRI in the Flatiron Health cohort was 4.4 months, median OS was 7.8 months, and longest time-on-treatment with zFOLFIRI was 266 days.

**Conclusions:** In these small real-world cohorts, clinical meaningful stable disease and overall survival on zFOLFIRI beyond progression on bevacizumab+FOLFIRI was observed in patients with mCRC. Further exploration of this approach is warranted.

## Introduction

Colorectal cancer is the third most common cancer diagnosed in the USA with an estimated 97,220 new cases of colon cancer and 43,030 cases of rectal cancer in 2018. It is also the third leading cause of cancer-related death among both men and women killing an estimated 50,630 people this year alone (1).

5-fluoruracil, leucovorin, and oxaliplatin (FOLFOX) or 5-fluorouracil, leucovorin, and irinotecan (FOLFIRI) remain standard front-line therapy backbones for patients with metastatic colorectal cancer (mCRC); (NCCN guidelines Version 2.2018). Hurwitz and colleagues first showed in 2004 that the addition of bevacizumab to fluorouracil-based combination chemotherapy significantly prolonged overall survival in patients with mCRC (2). The addition of anti-angiogenic therapy with bevacizumab thus became an acceptable component of standard of care in the frontline setting for patients with metastatic disease (3, 4).

After progressing on FOLFOX or FOLFIRI plus bevacizumab in the first line, the randomized phase III TML study (ML18147) demonstrated an overall survival benefit in the second line with the use of bevacizumab plus FOLFIRI vs. either FOLFIRI or FOLFOX alone, and patients frequently switched from FOLFOX to FOLFIRI or vice versa while continuing on bevacizumab (5). More recently, other antiangiogenic agents have been developed including large molecules such as ziv-aflibercept (Zaltrap or VEGF-TRAP) and ramucirumab (6). The RAISE study was a randomized double-blind multicenter phase III study of FOLFIRI plus ramucirumab or placebo in mCRC patients who progressed during or following first-line combination therapy with oxaliplatin, a fluoropyrimidine and bevacizumab and showed a 1.6 month increase in overall survival (11.7 months vs. 13.3 months) in the group treated with ramucirumab. Ramucirumab is approved in gastric, lung cancer and mCRC (7).

Aflibercept is a fusion protein containing key domains that recognize human VEGF receptors 1 and 2 and human IgG Fc (8). Ziv-aflibercept blocks all human VEGF-A isoforms, VEGF-B, and placental growth factor (PIGF). Known as “VEGF Trap,” ziv-aflibercept binds VEGF-A more tightly than native receptors. The randomized phase III VELOUR study (NCT00561470) investigated the use of ziv-aflibercept in combination with FOLFIRI (zFOLFIRI) in second line therapy for mCRC patients who previously failed prior oxaliplatin-containing therapy (9). VELOUR randomized ~600 metastatic colorectal cancer patients to each arm for treatment with aflibercept 4 mg/kg IV day 1 plus FOLFIRI every 2 weeks vs. placebo IV day 1 plus FOLFIRI every 2 weeks. Patients who received prior bevacizumab were eligible to enroll in the VELOUR study (9). VELOUR showed that overall survival (OS) was modestly prolonged in the study arm of zFOLFIRI vs. the placebo plus FOLFIRI arm. For the subgroup of patients who had received prior bevacizumab, OS was longer by 2.14 months for the zFOLFIRI vs. the placebo plus FOLFIRI arm (9).

zFOLFIRI was approved by the FDA in 2012 as a second line therapy for patients with mCRC. Current NCCN guidelines include the option to use zFOLFIRI in the second line for mCRC. However, the current NCCN guidelines (Version 2.2018) state that there are no data to suggest activity of zFOLFIRI in a patient who has progressed on FOLFIRI-bevacizumab, and that ziv-aflibercept has only shown activity when given in conjunction with FOLFIRI in FOLFIRI-naïve patients. Current 3rd line options for mCRC include regorafenib, based on the results of the CORRECT study in which a 1.4 month OS benefit was seen vs. best supportive care (BSC), or trifluridine-tipiracil which provided a 1.8 month OS benefit vs BSC in the refractory mCRC setting (10, 11). While additional therapies that target EGFR improve survival, these are only indicated for tumors with wild-type KRAS/NRAS genes and left-sided tumors. Clearly, options for patients who have failed initial therapy for metastatic disease are limited, especially for the subgroups with mutated KRAS or NRAS genes.

There have been no randomized comparisons of different anti-angiogenic agents such as ziv-aflibercept or ramucirumab plus combination therapy beyond first line bevacizumab-containing combinations with FOLFOX or FOLFIRI. Similarly, there are no randomized studies evaluating “second-line anti-angiogenic agents” such as ziv-aflibercept or ramucirumab in thesetting of bevacizumab use beyond progression in the second line setting where bevacizumab-containing combination therapies (after first-line bevacizumab-containing combination therapy in mCRC) are used.

Given the prior data that some patients who progressed on bevacizumab-containing combination therapy could derive some benefit from aflibercept plus FOLFIRI (VELOUR study) some patients in clinical practice have been offered such therapy beyond progression on bevacizumab containing combination therapy. While the original VELOUR study results suggested a modest benefit from the zFOLFIRI therapy beyond bevacizumab, there is little data on the use of zFOLFIRI beyond progression on bevacizumab-containing combination therapy regimens in general in mCRC. We therefore explored the real-world treatment outcomes in two cohorts of patients with mCRC treated with aflibercept in this context. We have treated 18 mCRC patients with zFOLFIRI beyond progression on bevacizumab in the second line and have noted in some cases prolonged stable disease. A significant number of the patients had prior bevacizumab plus FOLFIRI (N = 17), a clinical setting not previously evaluated in VELOUR or other trials. This case series therefore aims to summarize the patient demographics and clinical outcomes in patients that were treated with zFOLFIRI beyond progression on a bevacizumab-containing regimen. We also describe a separate cohort of patients in the Flatiron Health Database treated with zFOLFIRI after FOLFOX+bevacizumab and FOLFIRI+bevacizumab with similar findings. Our results provide a rationale for future exploration of zFOLFIRI beyond progression on bevacizumab plus FOLFIRI and bevacizumab plus FOLFOX. In the absence of additional clinical trial data, our observed clinically meaningful stable disease on zFOLFIRI beyond progression on bevacizumab plus FOLFIRI in mCRC suggests a possible further line of therapy for patients with otherwise limited options.

## Patients and Methods

### Chart Review

A retrospective chart review was conducted after review and approval of a clinical protocol by both the Research Review Committee and the Institutional Review Board at Fox Chase Cancer Center. A cohort was identified of 19 stage IV patients (18 with mCRC) who received aflibercept plus FOLFIRI (zFOLFIRI) in a GI Oncology Clinic (W.S.E-D.) at Fox Chase Cancer Center during the period from 2014-2018. All collected data was de-identified.

Data was collected on patient demographics, the clinical setting in which zFOLFIRI was used, i.e. capturing prior lines of therapy, mutation status of the mCRC (KRAS/NRAS/BRAF/MSI), the duration of zFOLFIRI therapy, along with response data (progression free survival (PFS), response rate (RR), time on treatment, stable disease, progressive disease), changes in tumor marker carcinoembryonic antigen (CEA), and toxicities that were encountered in the course of therapy with zFOLFIRI. Serum CEA was measured over time while on treatment with zFOLFIRI. The CEA value measured at each time point was divided by the baseline measurement to calculate a ratio and then plotted on a graph of percent change from baseline over time. Patients were stratified by their progression free survival (less than or greater than 4 months). Disease progression was always determined radiographically at our tertiary care NCI-designated comprehensive cancer center as what would necessitate a change in therapy regimen in the heavily pretreated advanced mCRC population with limited life expectancy.

A secondary goal of our research was to examine available clinical outcomes in a nationwide real-world database. After obtaining Institutional Review Board approval, data were obtained from the Flatiron Health EHR-derived database, a database comprised of patient-level structured and unstructured data, curated via technology-enabled abstraction. These data represent a demographically and geographically diverse patient population derived from the electronic health records of 280 cancer clinics (~2.1 million patients with cancer in the United States; https://flatiron.com/real-world-evidence/, August 2018, mortality v2.0). Data provided to third parties were de-identified and provisions were in place to prevent re-identification in order to protect patients' confidentiality.

**Figure 1:**
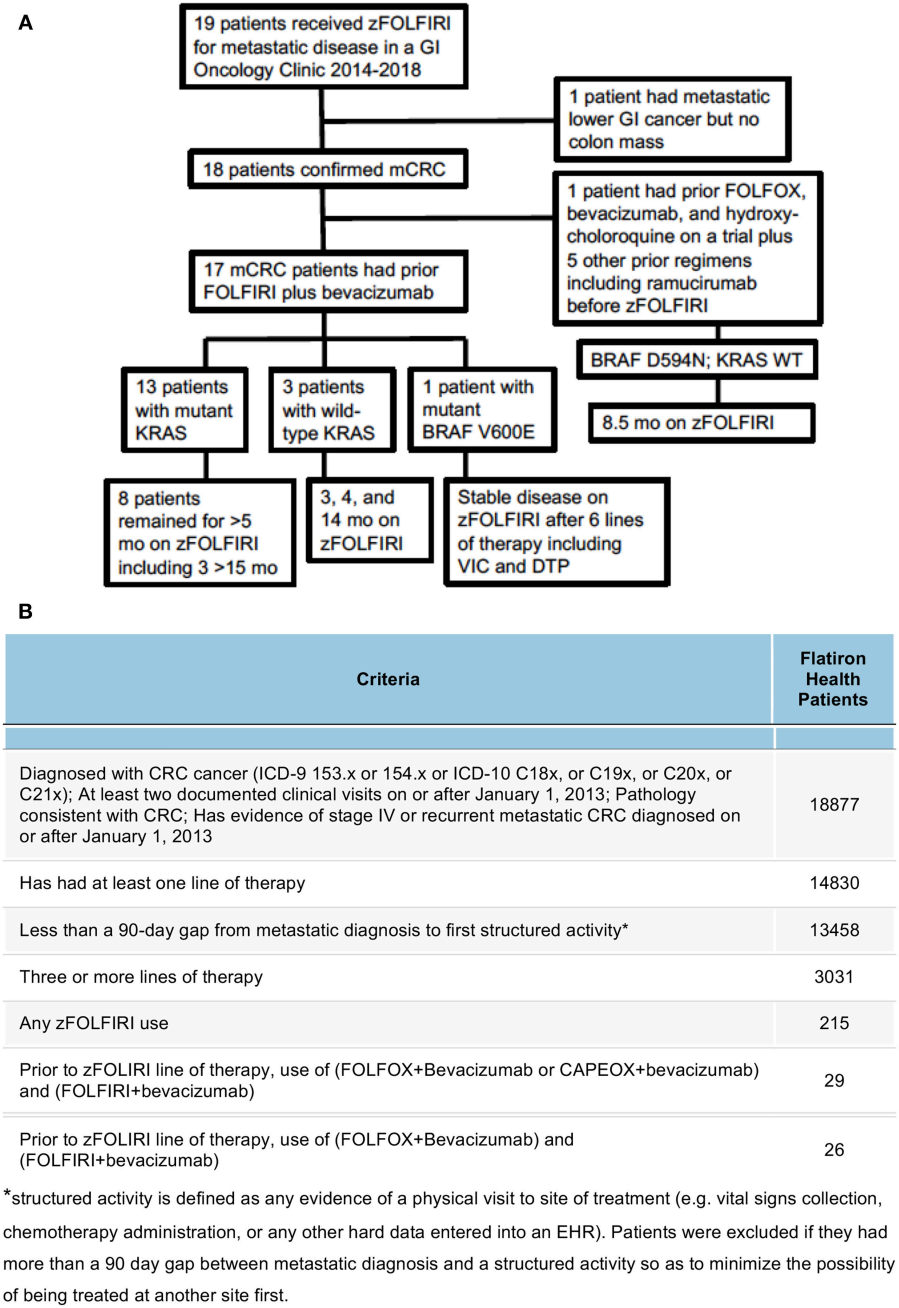
**(A)** Breakdown of the patient cohort treated with zFOLFIRI at Fox Chase Cancer Center leading to the 17 mCRC patients who had prior bevacizumab plus FOLFIRI. Details of tumor mutational status, some prior therapies, and disease control while on zFOLFIRI is shown. **(B)** Cohort selection of mCRC zFOLFIRI from patients in the Flatiron Health network. Out of the entire cohort of 18,877 patients diagnosed with CRC cancer, filters were applied to define a Flatiron Health cohort that most closely matched the FCCC cohort. Patients were filtered by increasingly strict criteria and excluded if they did not meet the criteria as defined in each row.

The study population in the Flatiron Health cohort included patients with an ICD code for colorectal cancer (ICD-9 153.x or 154.x or ICD-10 C18x, or C19x, or C20x, or C21x), at least two documented clinical visits on or after January 1, 2013, pathology consistent with CRC (confirmed via abstraction), had evidence of stage IV or recurrent mCRC diagnosed on or after January 1, 2013, who received zFOLFIRI after prior use of (FOLFOX+Bevacizumab or CAPEOX+bevacizumab) and FOLFIRI+bevacizumab (Figure 1B).

### Data Analysis

Given the pilot nature of this study, our emphasis was on collecting preliminary data, point estimation, and hypothesis generation to support future directions. We used standard descriptive statistics (e.g., means, medians, ranges, proportions, frequencies) to characterize the data collected. Overall survival (OS) and PFS time were characterized using the methods of Kaplan and Meier, and Fox Chase patients were analyzed separately from patients in the Flatiron Health cohort (12). OS time was defined as the number of days from first treatment with zFOLFIRI to death. PFS time was defined as the period from first treatment with zFOLFIRI to disease progression (determined by radiographic assessment in the FCCC cohort) or death, whichever occurred first. Patients who were alive, or alive and progression free at last contact were censored for OS and PFS analyses, respectively. Best response and treatment-related toxicities were characterized using frequency tables. A spider plot displaying the percent change in CEA from first measurement for each patient on the logarithmic scale was generated. All statistical analyses and plots were generated using the R statistical computing platform (https://www.r-project.org/).

To assess the relationship between rapid vs. prolonged progression free interval with changes in longitudinal CEA measures we first estimated the rate of change in CEA per day by fitting a separate linear regression for each patient using the data from the first 4 months on zFOLFIRI treatment. To adjust for substantial differences in baseline CEA values across patients, we first scale normalized the longitudinal measures for each patient by dividing by their CEA measurement closest to and prior to treatment initiation with zFOLFIRI. We used a Wilcoxon rank sum test (two-sided, α = 0.05) to compare slope estimates between early progressors and others.

## Results

### Patient Demographics, Tumor Characteristics and Prior Therapies

The median age at diagnosis of the cohort that received zFOLFIRI was 51 years of age (*N* = 19). This includes 12 females and 7 male patients. Of the 18 patients with mCRC, 8 patients had rectal cancer, 10 had colon cancer (9 with sigmoid or rectosigmoid) (Table 1). We also report the results of one patient who had a metastatic lower GI cancer without a colonic primary (Patient #17) who was treated with zFOLFIRI in the refractory setting. We included her in the descriptive analysis, but excluded her from the survival analysis. Of the 19 patients treated with zFOLFIRI, 8 had prior bevacizumab plus FOLFOX and 18 had prior bevacizumab plus FOLFIRI prior to receiving zFOLFIRI (Figure 1A). The median age at the time of zFOLFIRI treatment of the 18 mCRC patients (*N* = 18) was 54. The median number of systemic regimens prior to zFOLFIRI was 4 (range: 1–6). The full de-identified source patient data is provided as a Supplementary Table 1.

**Table 1:** Patient characteristics at baseline.

	**Fox chase cancer center zFOLFIRI cohort (*n* = 19)**	**Flatiron health zFOLFIRI cohort (*n* = 26)**	**CORRECT study (*n* = 505)**	**RECOURSE study (*n* = 534)**
Age at time of diagnosis (years), Median (Range)	51 (32–68)	61 (27–81)		
Age at start of zFOLFIRI (years), Median (Range)	54 (40–74)	62 (42–83)	61	63
**RACE**				
Caucasian	15 (79%)	18 (69%)	392 (78%)	306 (57%)
African american	1 (5%)	2 (8%)	6 (1%)	4 (< 1%)
Asian	1 (5%)	1 (4%)	76 (15%)	184 (34%)
Other/Not specified	2 (11%)	5 (19%)	31 (6%)	
**SEX**				
Male	7 (37%)	16 (62%)	311 (62%)	326 (61%)
Female	12 (63%)	10 (39%)	194 (38%)	209 (39%)
**PRIMARY SITE OF DISEASE**				
Colon	10 (53%)	22 (85%)	323 (64%)	338 (63%)
Rectum	8 (42%)	4 (15%)	151 (30%)	196 (37%)
Unknown	1 (5%)			
**MUTATIONAL STATUS^Σ^**				
KRAS mutated	13 (68%)	19 (73%)	205 (41%)	262 (59%)
KRAS wild type^#^	3 (16%)	3 (12%)	273 (54%)	272 (51%)
BRAF mutated	1 (5%)	1 (4%)	14 (4%)	
PIK3CA mutated^*^	2 (11%)			
TP53 mutated^*^	8 (42%)			

Σ *Patient with cancer of unknown primary excluded from mutational status*.

* *6 patients with missing data*.

# *4 patients in the Flatiron-Health Cohort with missing KRAS status*.

For the 17 mCRC patients who received zFOLFIRI after prior bevacizumab plus FOLFIRI, 13 had mutant tumor KRAS, 3 had WT KRAS, and one had BRAF V600E. For the single patient from the cohort of 18 mCRC patients who did not receive prior bevacizumab plus FOLFIRI, two VEGF-directed regimens were administered prior to zFOLFIRI, including FOLFOX plus bevacizumab as well as hydroxychloroquine as part of a clinical trial and irinotecan plus cetuximab plus ramucirumab as part of a separate clinical trial. 8 patients underwent a metastatectomy as part of their prior treatment (Table 2).

**Table 2:** Therapies given to mCRC patients prior to zFOLFIRI.

	**Fox chase cancer center zFOLFIRI cohort (*n* = 19)**	**Flatiron health zFOLFIRI cohort (*n* = 26)**	**CORRECT study (*n* = 505)**	**RECOURSE study (*n* = 534)**
Lines of therapy in metastatic setting prior to zFOLFIRI (number of lines), Median (Range)	4 (1–6)	3 (2–4)		
Number of previous anticancer therapies (on or after diagnosis of metastatic disease)				
1–2	8 (44%)	15 (58%)	135 (37%)	95 (18%)
3	4 (22%)	8 (31%)	125 (25%)	119 (22%)
≥4	6 (33%)	2 (8%)	245 (49%)	320 (60%)
No. patients who received FOLFOX plus bevacizumab	8 (44%)	26 (100%)^*^		
No. patients who received FOLFIRI plus bevacizumab	17 (94%)	26 (100%)^*^		
No. of patients who received cetuximab	5 (28%)	3 (12%)		
No. of patients who received trifluridine-tipiracil	3 (17%)	4 (15%)		
No. of patients who received regorafenib	1 (6%)	5 (19%)		
No. of patients treated with metatastectomy	8 (44%)			

* *These therapies were in the inclusion criteria for this cohort*.

**Figure 2:**
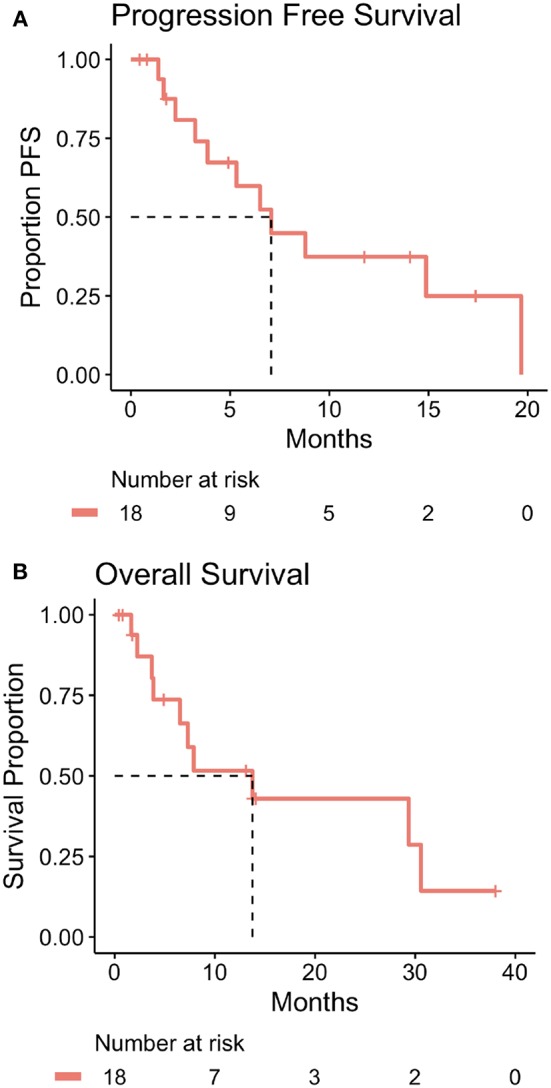
**(A)** Kaplan-Meier plot of progression free survival time for mCRC patients treated at Fox Chase Cancer Center with zFOLFIRI including table of number of patients at risk over time. The dotted line indicates the estimated median progression free survival time which is 7.1 months (214 days). Patient #17 was treated with zFOLFIRI but was excluded from the PFS, OS, and swimmer plot as they did not have documented mCRC. **(B)** Kaplan-Meier plot of overall survival time for mCRC patients treated at Fox Chase Cancer Center with zFOLFIRI including table of number of patients at risk over time. The dotted line indicates estimated median patient overall survival time which is 13.8 months (416 days). Patient #17 was treated with zFOLFIRI but was excluded from the PFS, OS, and swimmer plot as they did not have documented mCRC.

Overall, the average number of 2-week cycles of zFOLFIRI administered was 8.9 (range: 1–27 with 3 unknown precisely due to treatment primarily outside of our center and early disease progression). In patients treated at Fox Chase Cancer Center with mCRC, the median PFS while on zFOLFIRI was 7.1 months (214 days) and the OS from time of first treatment with zFOLFIRI was 13.8 months (416 days) (Figures 2A,2B).

**Figure 3:**
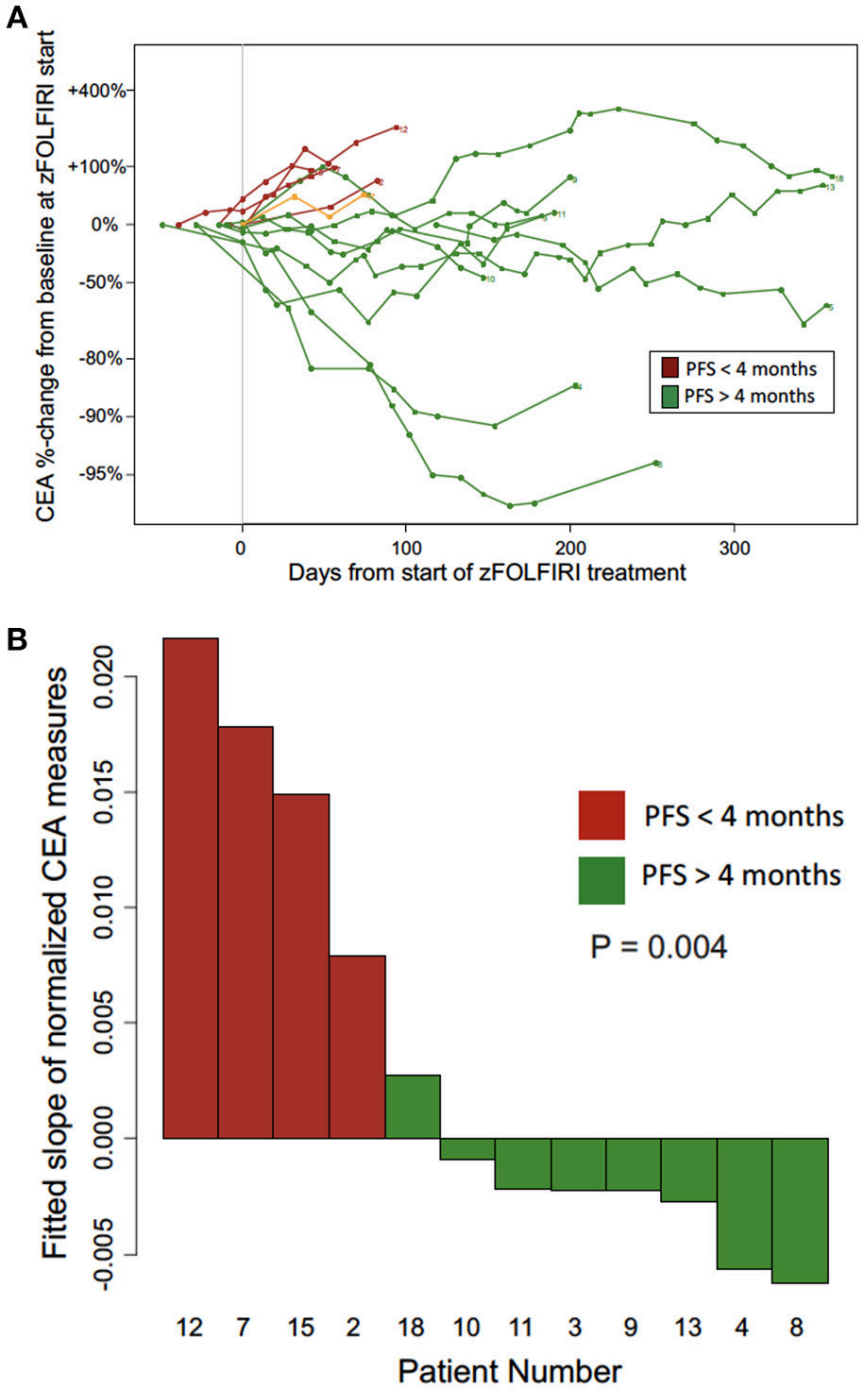
**(A)** CEA trend over time from the Fox Chase Cancer Center cohort treated with zFOLFIRI. The biochemical response to treatment with zFOLFIRI is shown. Serum levels of carcinoembryonic antigen (CEA) were measured over treatment time, and values are represented as percent change from baseline. Each line represents one patient. Patients were included if they had at least one scan to monitor treatment response after initiating treatment, had CEA values recorded in the electronic medical record, and either progressed or were followed for at least 120 days. Patients represented in red (n = 5) progressed in less than 4 months, and patients represented in green (n = 9) had a progression free survival of 4 months or more. Patient #17 was included in the spider plot (shown in orange) as the patient who had a lower GI malignancy did not have a documented colorectal cancer primary but did experience stable disease. Patient #17 who also had treated brain metastases requested discontinuation of therapy due to an overall poor quality of life and treatment-related toxicities such as nausea and diarrhea. **(B)** CEA trends among patients with early progression (PFS < 4 months) vs. longer disease control (PFS > 4 months) on zFOLFIRI (patients from Fox Chase Cancer Center cohort). The rate of change per day in normalized CEA measures over the first 4 months on zFOLFIRI treatment is shown for progressors (red) and other mCRC cases with PFS > 4 months (green).

**Figure 4:**
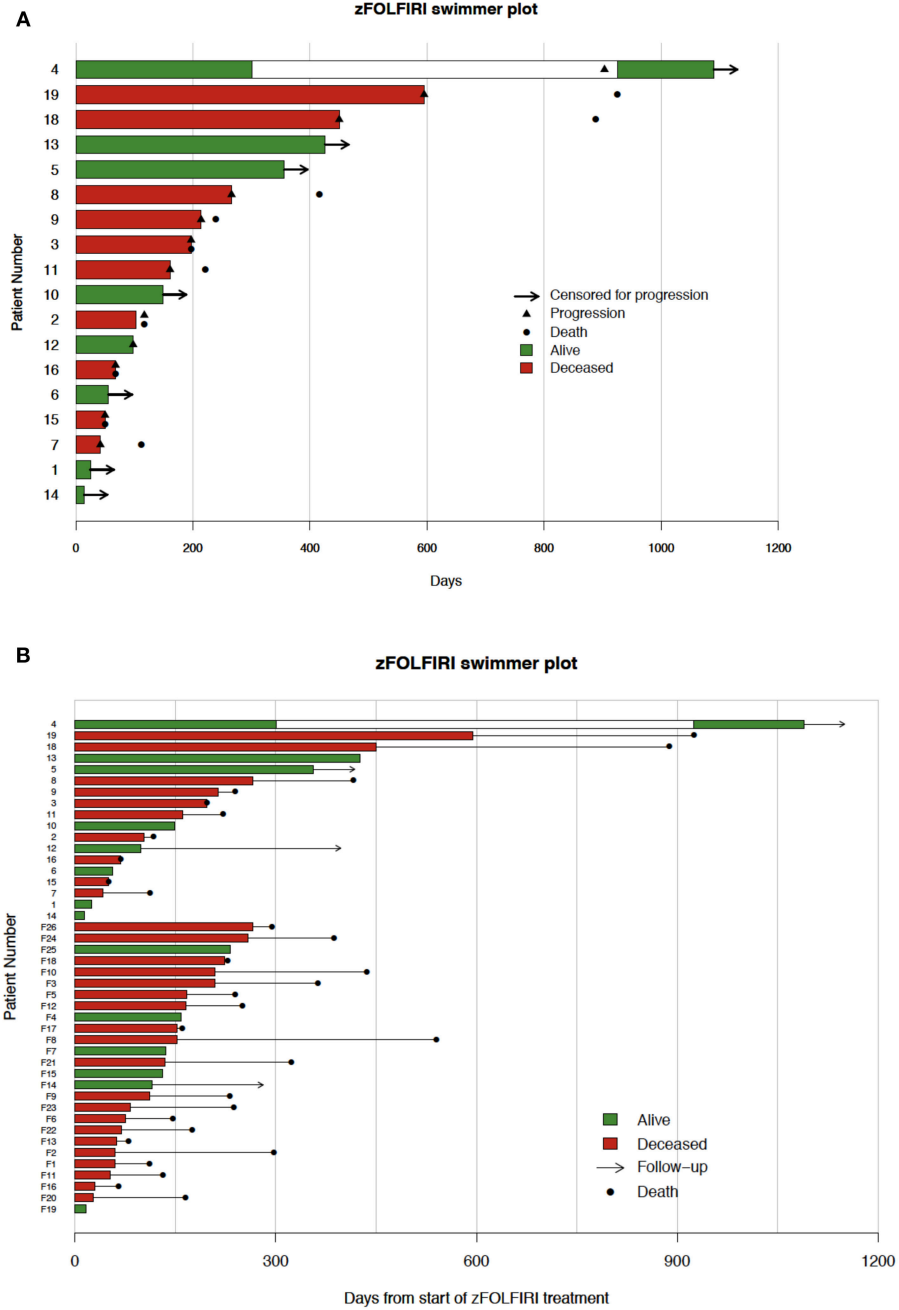
**(A)** Fox chase cohort swimmer plot. Each row represents one patient. Bar lengths show time on zFOLFIRI with green and red indicating live and deceased patients, respectively. Black triangles indicate the point of progression, and black dots indicate patient deaths. The white region for patient 4 marks a zFOLFIRI treatment holiday. Patient #17 was treated with zFOLFIRI but was excluded from the PFS, OS, and swimmer plot as they did not have documented mCRC. **(B)** Swimmer plot of both fox chase cancer center and flatiron health data. Each row represents one patient. Bar lengths show time on zFOLFIRI with green and red indicating live and deceased patients, respectively. Black dots indicate patient deaths, arrows indicate length of follow-up. The top 18 patients represent patients treated at Fox Chase Cancer Center. Numbers preceded by an F represent patients in the Flatiron Health Database.

The biochemical response to treatment with zFOLFIRI is shown in the Figure 3A. In patients who responded, CEA trends also tended to stabilize over time, correlating with stable disease in this cohort. Among the patients included in Figure 3A, the rate of change in normalized CEA measures over the first 4 months on zFOLFIRI treatment was significantly different (*p* = 0.004) between rapid progressors and other mCRC cases with PFS > 4 months (Figure 3B). All 4 early progressors had positive slopes as compared to only 1 of the mCRC patients with prolonged progression free survival intervals (Figure 3B). Some patients exhibited a durable response, with 3 patients who remained on therapy for >15 months (Figure 4A).

In the Flatiron Health cohort, 26 patients were identified as having received zFOLFIRI after previous lines of FOLFOX+bevacizumab and FOLFIRI+ bevacizumab (Table 1). The median age at time of treatment with zFOLFIRI in this cohort was 62 with an ECOG performance status of 0-2, and the majority of these patients had tumors that were KRAS-mutated (19/26). The median time-on-treatment for these patients was 4.4 months (132.5 days), and the median OS for these patients was 7.8 months. The longest duration of treatment with zFOLFIRI in this cohort was 266 days (Figure 4B). When combined with the FCCC cohort, the median time on treatment remained the same at 132.5 days.

### Description of Adverse Events in 18 mCRC Patient Cohort Treated With zFOLFIRI

zFOLFIRI was well tolerated although 8/18 patients (44%) experienced a grade 3 or higher toxicity, including GI-related events (ileus, small bowel obstruction, or fistula formation) in 16% of patients as well as neutropenic fever in 22% of patients (Table 3). 15/18 patients (83%) experienced a toxicity of any grade. Of note, the rate of neutropenic fever and GI events was higher in our cohort vs. the original VELOUR study (fistula formation was 1.1 vs. 5% in our cohort and febrile neutropenia was 6.5 vs. 22%) however our patient cohort was small and a more heavily pretreated.

**Table 3:** Adverse events in all patients treated at fox chase cancer center with zFOLFIRI.

**Grade 3 toxicities**	No. of patients^*^	**Grade 2 toxicities**	**No. of patients**	**Grade 1 toxicities**	**No. of patients**
Neutropenic fever	4	fatigue	1	Hyperbilirubinemia	1
Ileus/Small Bowel Obstruction	2	TIA	1	Diarrhea	1
Fistula formation	1	Nausea/Vomiting	1	Myalgia	1
Syncope	1				

* *1 patient experienced more than 1 grade 3 toxicity*.

### Description of Specific Cases of Interest Treated With zFOLFIRI Within the 18-Patient mCRC Cohort

#### Patient 4; Mutated KRAS, Peritoneal Spread, Prior FOLFIRI Plus Bevacizumab, zFOLFIRI PFS > 1.5 year

Patient is a 43-year-old woman with KRAS mutated (G12V) metastatic mucinous adenocarcinoma who was first diagnosed at age 36 with widespread peritoneal disease. She subsequently underwent a debulking surgery followed by 4 cycles of FOLFOX-based chemotherapy, and then further debulking and hyperthermic chemotherapy (HIPEC) instillation. She completed a further 8 cycles of palliative FOLFOX with disease control for approximately 9 months. When her disease subsequently progressed, she was treated with FOLFIRI plus bevacizumab but suffered severe diarrhea requiring cessation of 5-FU at the time. After suffering a pulmonary embolism, irinotecan plus bevacizumab were stopped and the patient was treated with another round of cytoreductive surgery plus mitomycin-C based HIPEC.

Her disease remained stable for a subsequent 2 years until progression was noted on a PET-CT scan at which point she was started on zFOLFIRI. The patient stayed on therapy despite requiring a dose reduction for approximately 9 months after which time her scan showed stable disease. The decision was then made to treat her dominant pelvic mass with palliative XRT and she subsequently went onto maintenance capecitabine with the addition of bevacizumab for 17 months. Upon disease progression at that time, zFOLFIRI was restarted with stable disease for an additional 8 months with a continued response at the time of data censorship. The CEA trends observed in this patient showed reduction during both zFOLFIRI treatment periods (Supplementary Figure 1).

#### Patient 5; KRAS G12C Mutant, Prior FOLFIRI Plus Bevacizumab, zFOLFIRI PFS ~1 Year

A 56-year-old man was diagnosed with KRAS G12C mutated stage IIIB rectosigmoid adenocarcinoma. Post-resection he was treated with adjuvant FOLFOX. 1.5 years later his disease recurred in the lungs and after a metastatectomy he was treated palliatively with FOLFIRI and bevacizumab for 11 months. At the point of disease progression, he was started on zFOLFIRI which he took for about 2 months. However, due to personal issues, the patient was lost to follow-up for roughly 3 months.

When he again presented to our clinic to reinitiate therapy, restaging scans taken at that time showed stable disease and he resumed treatment without issue. He remained on therapy for an additional 10 months after which he was lost to follow-up. His most recent restaging scans prior to him leaving our clinic showed ongoing disease stability.

#### Patient 8; BRAF D594N Mutant, Prior Bevacizumab, Ramucirumab and Cetuximab, zFOLFIRI PFS ~1 year

A 42-year-old man was diagnosed with KRAS wild-type and BRAF D594N mutated stage III rectal carcinoma. He was started on capecitabine, oxaliplatin, and radiation and subsequently underwent an abdominal perineal resection followed by adjuvant capecitabine and oxaliplatin (XELOX) chemotherapy. After 1 year, the patient recurred with disease in the lungs, liver, and lymph nodes. He was started on a phase II trial of FOLFOX, bevacizumab and hydroxychloroquine but stopped after 6 months due to an oxaliplatin reaction and was treated with maintenance 5-FU plus bevacizumab and hydroxychloroquine.

His disease remained stable for approximately 18 months until he developed progressive disease in the lungs and liver, both of which were resected. However, his disease recurred in the liver, lungs, and retroperitoneum 4 months later and he was started on cetuximab, irinotecan, and ramucirumab on a separate clinical trial but disease unfortunately progressed after 2 months. He was then treated with trifluridine-tipiracil for roughly 1 year after which his disease progressed and he was started on zFOLFIRI. He had a grade 3 small bowel obstruction but once this resolved was able to stay on zFOLFIRI for 8 months prior to progression of disease. The patient survived for another 13.8 months from the time of initiation of zFOLFIRI.

#### Patient 10; BRAF v600E Mutated, Prior FOLFIRI Bevacizumab and EGFR Directed Therapy With VIC and DTP, zFOLFIRI PFS Ongoing at > 4 Months

A 44-year-old woman was diagnosed with sigmoid colonic adenocarcinoma with mesenteric adenopathy on CT scan, hemicolectomy revealed a stage IIIB tumor (T3N1M0) that was moderately differentiated. Post-resection, the patient was treated with adjuvant FOLFOX for 6 months and entered surveillance. Three years later her CEA began to rise with CT scan revealing new bilateral ovarian metastases which were biopsy proven as metastatic colonic adenocarcinoma. Analysis at that time was significant for a tumor BRAF V600E mutation. She was then treated with FOLFIRI for 4 cycles initially with bevacizumab, however due to delayed wound healing bevacizumab was held.

Restaging scans after 4 cycles showed progression of disease and therefore she was treated for 4 months with FOLFIRI and cetuximab, however her disease then continued to grow. Given her BRAF V600E mutation, she was treated with vemurafenib, irinotecan, and cetuximab (VIC) (13) with disease control for 6 months. She was then treated with dabrafenib, trametinib, and panitumumab (DTP) due data showing effectiveness of this combination in these patients (14), however she progressed after 3 months. Given that she had never progressed on bevacizumab, she restarted FOLFIRI and bevacizumab which controlled disease for an additional 3 months. At time of progression, she was consented to zFOLFIRI. Despite grade 1 diarrhea, nausea, and vomiting she has tolerated therapy well and has ongoing stable disease at >4 months into treatment at time of censorship.

#### Patient 13; KRAS Wild-Type, Prior FOLFIRI Bevacizumab and zFOLFIRI PFS >14 Months

A 68-year-old man was first diagnosed with metastatic KRAS wild-type rectal cancer at the age of 65 and was treated with FOLFOX plus bevacizumab for 5 months and upon progression was treated with FOLFIRI plus cetuximab for a subsequent 8 months. He then underwent a resection of liver metastases and received radiation to the pelvic region for pain control. He then was continued on maintenance 5-fluorouracil plus capecitabine-based chemotherapy for 5 months after which time his cancer progressed and a liquid biopsy at that time revealed a KRAS Q61H mutation which was felt to be acquired from prior therapy with cetuximab. He was therefore consented to FOLFIRI plus bevacizumab. After 5 months of treatment on this regimen his cancer again progressed and he was consented to zFOLFIRI. On the zFOLFIRI regimen, the patient's disease initially regressed in the liver and then remained stable on multiple scans, allowing the patient to remain on treatment for 14 months with an ongoing response at time of data censorship.

#### Patient 18; KRAS Mutant, Prior FOLFIRI Plus Bevacizumab, zFOLFIRI PFS ~18 Months

A 52-year-old woman was diagnosed with stage IV KRAS G12C mutated rectosigmoid colonic adenocarcinoma involving the liver and was started on treatment with capecitabine, oxaliplatin, and bevacizumab. She was treated for 6 months with treatment response and subsequently underwent a liver-directed metastatectomy followed by resection of the primary tumor via a low anterior resection. She was then started on FOLFIRI plus bevacizumab after imaging showed progression of disease in the liver on which she was maintained for 4 months. At the time of disease progression, she was started on zFOLFIRI which showed a partial response in the liver. The patient was ultimately able to stay on this treatment for 18 months before imaging showed progression of her liver disease requiring cessation of this line of therapy. The patient ultimately expired 30 months after starting treatment with zFOLFIRI.

## Discussion

Our results identify the potential for clinical benefit in two cohorts of advanced mCRC patients (a Fox Chase Cancer Center cohort, and a Flatiron Health EHR-derived cohort who received zFOLFIRI after prior bevacizumab+FOLFOX and bevacizumab+FOLFIRI). The FCCC cohort was a series of advanced mCRC patients (*N* = 18) who received zFOLFIRI in the third line after progression on FOLFIRI plus bevacizumab (*N* = 17) given as second line therapy. This is typically a population of patients with limited options and most of these patients had tumors that were KRAS-mutated, making their therapy options even more limited. The Flatiron Health cohort included 26 patients who received zFOLFIRI after prior bevacizumab+FOLFOX and bevacizumab+FOLFIRI. The latest available NCCN guidelines (Version 2.2018) state that there are no data to suggest activity of zFOLFIRI in a patient who has progressed on FOLFIRI-bevacizumab, and that ziv-aflibercept has only shown activity when given in conjunction with FOLFIRI in FOLFIRI-naïve patients. Our results in the combined population of 45 patients with advanced mCRC suggest potential for significant disease control with zFOLFIRI beyond prior FOLFIRI plus bevacizumab. CEA is often used to track response to treatment in patients with mCRC, with stabilization or a decline in CEA values correlating with response to treatment. In the FCCC cohort of patients treated with zFOLFIRI who had stable disease at the time of their first imaging, the CEA trend stabilized as would be expected. In patients who did not respond, the CEA predictably increased as illustrated in Figure 3 for this cohort of patients. For the initial cohort of FCCC patients, PFS was extended by 5 months or greater in the majority of patients, and a subgroup of patients (23.5%) who experienced prolonged stable disease of greater than 1 year indicating significant activity of zFOLFIRI in this population.

While our patient cohort was slightly younger with a median age of 54 at time of zFOLFIRI vs. 61 in the VELOUR study, they were more heavily pretreated and had more refractory disease, and so their younger age may be counterbalanced by the advanced nature of their disease (9). For the 13 patients with mutated KRAS, 8 patients remained on zFOLFIRI for >5 months including 3 patients who remained on therapy for >15 months. The median PFS was 7.1 months (214 days) and the median OS was 13.8 months (416 days). The PFS prolongation compares favorably with the 2 month PFS prolongation observed in third line therapy with either regorafenib or trifluridine-tipiracil (10, 11).

To add to the FCCC cohort, we also explored clinical outcomes data from the national Flatiron Health EHR-derived database, a real-world data set which represents a nationwide sample from community and academic practices in the United States. From the Flatiron Health database we identified a cohort of 26 additional patients treated with zFOLFIRI after receiving FOLFOX and bevacizumab as well as FOLFIRI and bevacizumab. These patients had a median time on treatment of 4.4 months and an overall survival of 7.8 months. While overall survival of the cohort from the Flatiron Health database was less than in the Fox Chase patient cohort, the time on treatment was still longer than the historic PFS of either regorafenib or trifluridine-tipiracil, each of which have a PFS of roughly 2 months. The fact that median age at time of zFOLFIRI treatment in the Flatiron Health cohort was 62, 8 years older than in the Fox Chase Cancer Center cohort, may explain the shortened overall survival although despite this difference in median age, OS was still longer than that reported in other nth line studies. When combined with our cohort, the median time-on-treatment was 132.5 days and did not change significantly. Whether median age at time of treatment impacts PFS or OS was not a goal of our study and would need to be investigated in the future.

Interestingly, 3 additional patients treated with capecitabine, oxaliplatin, and bevacizumab and also treated FOLFIRI with bevacizumab before zFOLFIRI were identified in the Flatiron Health database. One of these patients remained on zFOLFIRI for 20.5 months, indicating that this regimen may be effective, in the patients who experience prolonged disease control, regardless of the previous specific fluoropyrimidine used. Our combined data therefore lends support to our hypothesis that zFOLFIRI has activity in the refractory setting beyond FOLFIRI and bevacizumab and prior oxaliplatin-based combination chemotherapy plus bevacizumab.

Our goal was to test the hypothesis that ziv-aflibercept, which has a broad and potent anti-angiogenic activity, may be active beyond progression on bevacizumab (anti-VEGF) combination therapy in later lines of mCRC therapy and may offer a clinical benefit to patients while maintaining a similar toxicity profile to their previous bevacizumab-containing therapy. The finding of disease control with zFOLFIRI despite prior bevacizumab plus FOLFIRI suggests that ziv-aflibercept at least in combination with FOLFIRI has potential for clinical activity beyond bevacizumab. The results also suggest the clinical activity of ziv-aflibercept in combination with FOLFIRI can be observed beyond prior FOLFIRI including prior FOLFIRI plus bevacizumab.

Vascular endothelial growth factor (VEGF) is a major regulator of tumor angiogenesis (15) and a target for anti-cancer therapy development (16). Previous studies have shown that plasma VEGF levels increase after intravenous administration of bevacizumab (17). However, these are acute changes and the increase in VEGF may be due to impaired clearance (18). The use of VEGF as a predictive biomarker for bevacizumab therapy has remained inconclusive, however VEGF is a potent mediator of tumor angiogenesis and higher levels may be indicative of increased tumor angiogenesis and increased growth (19). Ziv-aflibercept is a more potent inhibitor of VEGF as compared to bevacizumab (20). As such, the question arises as to whether the disease control observed within our cohort with zFOLFIRI may be due to the more potent inhibition of VEGF signaling by ziv-aflibercept. On the other hand, other proangiogenic factors such as VEGF-C, PIGF or VEGF-D may contribute to the emergence of resistance to anti-VEGF therapy with bevacizumab and may in part explain subsequent sensitivity to ziv-aflibercept after progression (21).

In addition to the observed prolonged disease control in the FOLFIRI plus bevacizumab pretreated advanced mCRC population, it is noteworthy that one of our patients had an aggressive BRAF V600E mutated mCRC that achieved disease control with zFOLFIRI after prior sequential VIC (13) and DTP (14) targeted regimens, as well as multiple prior treatments. The setting in which zFOLFIRI was used in this patient represents a currently difficult clinical situation with a subset of mutant BRAF mCRC patients who have a very poor prognosis and extremely limited treatment options especially beyond the targeted therapy combinations.

We believe a strength of our report is that the population of mCRC patients from our Fox Chase Cancer Center case series that we treated is typical of the population of advanced mCRC patients treated at a tertiary care NCI-designated comprehensive cancer center. We note that the population we treated with zFOLFIRI at Fox Chase Cancer Center and the Flatiron Health database cohort with advanced mCRC treated with zFOLFIRI were both enriched for patients with aggressive refractory KRAS-mutant mCRC which currently presents a major challenge in clinical practice. The common theme in our Fox Chase Cancer Center cohort is that all 19 patients were heavily pretreated with prior chemotherapy regimens, 18 of the 19 patients had mCRC, and 17 of the 18 mCRC patients had prior FOLFIRI plus bevacizumab, a setting not predicted to respond to zFOLFIRI and not currently recommended for such treatment in the 3rd line setting. The Flatiron Health database-derived cohort with the observed outcomes further supports this notion. While future larger cohort studies and randomized trials can investigate in more detail the impact of genetic subtypes of mCRC, what is clear from our case series is that patients with various genetic subtypes were found to have disease control with zFOLFIRI despite prior therapies, and some in each subgroup had prolonged disease control. It will be important in the future to further investigate the basis for more durable disease control, and characterize the influence of underlying tumor biology and clinical natural history.

A number of mechanisms of resistance to antiangiogenic therapy have been recognized (22). These include growth factor redundancy, effects of bone marrow-derived cells, stromal cells, vasculogenic mimicry, and a number of emerging molecular mechanisms or factors such as glycosylation, extracellular vesicles, or polymorphisms (22). A number of biomarkers have been identified and associated with prolonged OS on zFOLFIRI (23). Yoshino et al. found that the baseline levels of 8 biomarkers (TIMP-1, IL-8, EN-RAGE, SP-D, TN-C, IGFBP-1, Kallikrein 5, TNFR2) correlated with OS. This list is only partially overlapping with a prior study that identified the baseline levels of six markers (CRP, Gro-alpha, IGFBP-1, TF, ICAM-1 and TSP-2) correlated with PFS and OS after bevacizumab and everolimus (24). A prior study noted that Ras mutated as well as BRAF mutated mCRC can respond to zFOLFIRI (25) while another study identified SNPs in VEGFB as correlated with PFS and high IL8 with worse PFS (26). miR-21 has been found to be increased after bevacizumab therapy suggesting more complex interactions in signaling that may inform biomarker choice for efficacy or resistance to anti-angiogenic therapy (27).

zFOLFIRI was well tolerated in our patient cohort. ~83% of patients treated with zFOLFIRI in the original VELOUR study experienced a grade 3 or 4 adverse event, including VEGF-specific events [i.e., arterial (1.8%) or venous (7.9%) thromboembolism, hemorrhage (2.9%), or hypertension (19%)] as well as chemotherapy related events, such as diarrhea (19%), thrombocytopenia (3.3%) and febrile neutropenia (5.7%) (9). In our patients 44% experienced a grade 3 adverse event, including GI-related events and neutropenic fever. While rates of some of these events were somewhat higher in our cohort vs. the original VELOUR study (febrile neutropenia: 6.5 vs. 22%, GI fistula formation:1.1 vs. 5% in our cohort), our patient cohort was small making absolute percentages difficult to interpret. They were also more heavily pretreated and thus may have been more prone to adverse events. Of note, these adverse events are in line with the original studies of bevacizumab in combination with chemotherapy. In the original trial by Hurwitz, et al. establishing bevacizumab as an active agent in colorectal cancer, 84.9% of patients experienced a ≥ grade 3 adverse event, including thrombotic events (19.4%), hemorrhage (3.1%), and hypertension (11%), similar to the rates in the VELOUR study and greater than rates of some of these toxicities seen in our cohort (2). Similarly, the RAISE trial of FOLFIRI-ramucirumab after treatment with oxaliplatin-based chemotherapy yielded a 79% rate of grade 3 or higher adverse events (7). Rates of febrile neutropenia among these trials are overall quite low, with 3% in the RAISE study and 2.9% in the VELOUR trial. Given the increased potency of ziv-aflibercept vs. bevacizumab or ramucirumab, efficacy does not seem to be compromised by increased toxicity (8).

Similarly, the rate of grade 3 adverse events in our cohort is in line with studies of other non-VEGF-based therapies conducted in patients with refractory mCRC. For example, in the RECOURSE study which led to the approval of trifluridine-tipiracil in this setting, 69% of patients experienced a grade 3 or higher adverse event (10). In the CORRECT trial of regorafenib vs. placebo for patients with refractory mCRC, 54% of patients had a ≥ grade 3 toxicity (10). Therefore, the rate of adverse events seen in our patient cohort is not unexpected or prohibitive.

Our results support the additional exploration of clinical activity of zFOLFIRI in later lines of therapy, in additional retrospective and exploratory prospective cohorts. Efficacy beyond FOLFIRI plus bevacizumab in addition to oxaliplatin-based combination chemotherapy plus bevacizumab is the mCRC population of interest. Patients with mutant KRAS are of interest and predicted to have potential for benefit based on our observations, although we also saw prolonged time to disease progression in other genetic subtypes such mutant BRAF, or wild-type KRAS and BRAF. In the absence of a clinical trial, our results support the feasibility and provide some indication of expected outcomes for zFOLFIRI in patients with advanced mCRC including those who have received bevacizumab plus FOLFIRI in the second line or later therapy settings in addition to oxaliplatin-based combination chemotherapy plus bevacizumab. Future studies will need to incorporate appropriate biomarkers and consider disease control as a clinically meaningful endpoint for this patient population.

It is important to note that the zFOLFIRI regimen beyond FOLFIRI plus bevacizumab was associated with disease control for over 1 year in several patients. We recognize that the PFS of 7.1 months with zFOLFIRI in the 3rd line beyond FOLFIRI plus bevacizumab is not significantly different from the PFS of 6.9 months with zFOLFIRI in the second line observed in the VELOUR study. We are certainly reassured that it is very similar despite the fact that 17 of the 18 mCRC patients treated with zFOLFIRI had prior FOLFIRI plus bevacizumab. The lesser PFS prolongation with zFOLFIRI observed in the Flatiron Health database-derived cohort is a real-world experience from a second older patient cohort and still supports our overall conclusions regarding potential benefit for zFOLFIRI beyond both FOLFOX plus bevacizumab and FOLFIRI plus bevacizumab. Both the Fox Chase Cancer Center and the Flatiron Health database-derived cohort outcomes with zFOLFIRI suggest benefit from zFOLFIRI in patients who were previously treated with bevacizumab plus FOLFIRI in addition to other regimens, and those outcomes were better than what has been observed with current standard of care such as regorafenib or trifluridine-tipiracil. It is noteworthy that both our Fox Chase Cancer Center cohort and the Flatiron Health database-derived cohort were enriched for patients with KRAS-mutant mCRC, but that within our Fox Chase Cancer Center cohort, disease control was also observed with wild-type KRAS/NRAS or mutant BRAF. Future studies can focus more on the impact of driver mutations such as KRAS/NRAS or BRAF on disease control with zFOLFIRI.

In summary, we observed meaningful stable disease on zFOLFIRI beyond progression on bevacizumab plus FOLFIRI in a total of 44 mCRC patients from two real-world cohorts (Fox Chase Cancer Center as well as the Flatiron Health database) suggesting that additional exploration of the clinical benefit of this therapeutic approach is warranted. An appropriate next step could be a comparative effectiveness study of zFOLFIRI vs. other third-line treatments in real-world retrospective cohorts or perhaps a prospective pragmatic clinical trial.

## Data Availability

All datasets generated for this study are included in the manuscript and/or the supplementary files.

## Author Contributions

All authors listed have made a substantial, direct and intellectual contribution to the work, and approved it for publication.

### Conflict of Interest Statement

BA and NM are employees of Flatiron Health. The remaining authors declare that the research was conducted in the absence of any commercial or financial relationships that could be construed as a potential conflict of interest.

## Supplementary Material

Supplementary Figure 1 CEA trends in Fox Chase Cancer Center patient #4 are shown during the time the patient was treated with zFOLFIRI. The grey region represents time off of zFOLFIRI when the patient was treated with maintenance capecitabine.

Supplementary Table 1 De-identified patient data for 19 patients treated at Fox Chase Cancer Center with zFOLFIRI.
