# Supplementary material for: Disease Control With FOLFIRI Plus Ziv-aflibercept (zFOLFIRI) Beyond FOLFIRI Plus Bevacizumab: Case Series in Metastatic Colorectal Cancer (mCRC)
Source: Front Oncol. 2019 Mar 14;9:142. doi: 10.3389/fonc.2019.00142 (PMC6426764; doi:10.3389/fonc.2019.00142)

**Supplementary Figure 1. CEA trends in Fox Chase Cancer Center patient #4 are shown during the time the patient was treated with zFOLFIRI.** The grey region represents time off of zFOLFIRI when the patient was treated with maintenance capecitabine.


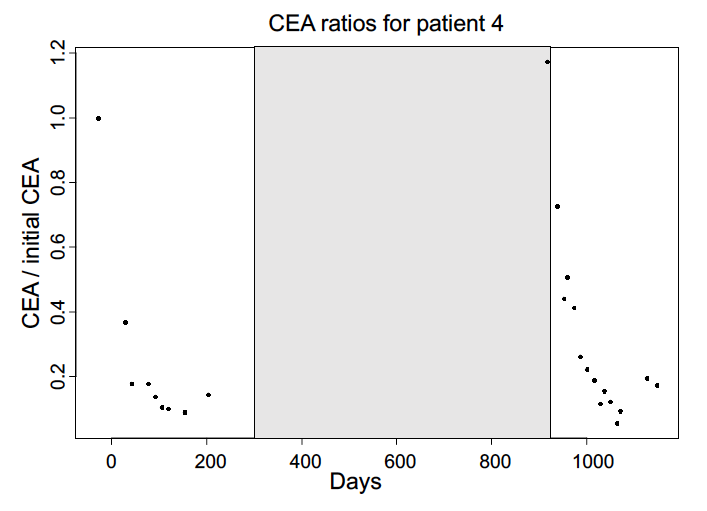

Supplement: Supplementary Figure 1 — CEA trends in Fox Chase Cancer Center patient #4 are shown during the time the patient was treated with zFOLFIRI. The grey region represents time off of zFOLFIRI when the patient was treated with maintenance capecitabine. [file Data_Sheet_1.docx]
